# Cover Image: “The POCUS Lesson of Professor Tulp”

**DOI:** 10.24908/pocus.v6i1.14764

**Published:** 2021-04-22

**Authors:** James S Newman

**Affiliations:** 1 Mayo Clinic, Hospital Medicine, Rochester Hospital Operation Command Center (RHOCC)

**Keywords:** POCUS, art

## POCUS Arts and Humanities

**Figure 1:**
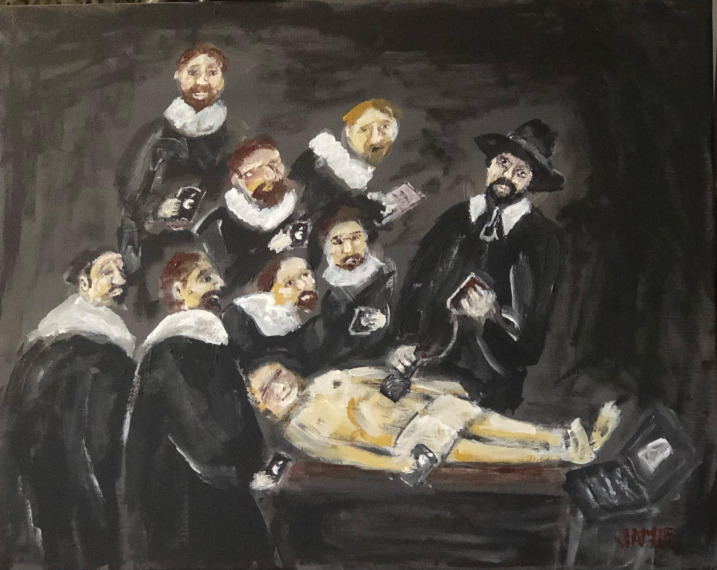
An homage to Rembrandt’s “The Anatomy Lesson of Dr. Nicolaes Tulp” for the modern physician (Acrylic on canvas).

